# Spinal Epidural Abscess

**DOI:** 10.21980/J8T938

**Published:** 2020-01-15

**Authors:** Christine T Luo, Jennifer Yee

**Affiliations:** * The Ohio State University, Department of Emergency Medicine, Columbus OH

## Abstract

**Audience:**

The aim of this simulation case is to educate senior medical students, resident physicians, and advanced practice providers on the recognition, diagnosis, and management of spinal epidural abscesses. This scenario is most applicable to the emergency medicine setting but can be applied to the outpatient office or urgent care settings.

**Introduction:**

Spinal epidural abscess is an infection leading to an epidural collection of purulent material. This uncommon condition is estimated to occur less than 12 times per 100,000 hospital admissions.^1^,^2^ However, this infection can lead to devastating neurological sequelae via cord compression, spinal vascular interruption, and inflammatory etiologies;^3^,^4^ thus, prompt diagnosis is essential. Unfortunately, spinal epidural abscesses may be difficult to identify clinically due to variable clinical presentations. The goal of this scenario is to increase awareness of this critical diagnosis.

Detailed history-taking to identify risk factors will aid in the recognition of spinal epidural abscesses. Many of the risk factors are related to increased infectious risk from hematogenous spread, iatrogenic inoculation, or direct extension.^1^ Individuals with conditions including intravenous (IV) drug use, alcohol abuse, diabetes, human immunodeficiency virus (HIV), cancer, hepatic disease, renal disease, and other immunocompromising conditions are at increased risk of developing epidural abscesses.^1^ Primary infectious sources include dental abscesses, endocarditis, vertebral osteomyelitis, and soft tissue infections. Spinal procedures including spinal surgeries, paraspinal injections, and placement of epidural catheters or stimulators can also predispose to infection.^2^,^4^

Classic symptoms for spinal epidural abscesses include fever, back pain and neurological changes.^1^,^5^ Back pain is the most frequent presenting symptom, occurring about 70%–90% of the time.^1^ However, fever is the least frequent presenting symptom^4^ and neurological findings only occur in about one-third of cases.^2^ Neurological symptoms include motor weakness, sensory changes, urinary retention, overflow urinary incontinence, bowel dysfunction, hyperreflexia, radicular pain, spinal shock or cauda equina syndrome.^1^,^4^

Laboratory findings may include systemic leukocytosis and elevated inflammatory markers. Whereas leukocytosis is estimated to be present in two-thirds of cases,^2^ Davis, et al. showed that with the concurrent presence of a risk factor, an elevated erythrocyte sedimentation rate (ESR) had 100% sensitivity and 67% specificity for spinal epidural abscesses.^5^

Magnetic resonance imaging (MRI) with gadolinium contrast is the preferred imaging modality for diagnosing spinal epidural abscesses. Computed tomography (CT) with myelography can be considered if MRI is contraindicated.^1^ Given that abscesses may be multifocal, further spinal imaging beyond a single spinal segment should be considered during evaluation. Lumbar puncture is not recommended due to risk of iatrogenic infectious spread.

Treatment of epidural abscesses includes obtaining blood cultures and prompt antibiotic administration with early surgical evaluation to determine if operative intervention is warranted. *Staphylococcus aureus* is the most common microbial cause, contributing to about two-thirds of cases.^3^,^4^ Other microbial causes include coagulase-negative *Staphylococcus* (ie, *Staphylococcus epidermidis*), *Streptococcus*, gram-negative bacilli (ie, *Pseudomonas aeruginosa* and *E. coli*), and less commonly, anaerobic bacteria, fungi, mycobacteria and parasites.^1^,^2^ Empiric antibiotic treatments generally include vancomycin and a third- or fourth- generation cephalosporin.^2^,^4^

This simulation session will highlight the importance of recognizing and aggressively treating this uncommon but potentially devastating condition.

**Educational Objectives:**

After this simulation case, learners will be able to diagnose and manage patients with spinal epidural abscesses. Specifically, learners will be able to:

1. Obtain a detailed history, including past infectious, surgical, procedural and social history to evaluate for epidural abscess risk factors. Describe clinical signs and symptoms of spinal epidural abscesses and understand that initial clinical presentations can be variable.
2. Perform a focused neurological exam including evaluation of motor, sensory, reflexes, and rectal tone.
3. Order appropriate laboratory testing and imaging modalities for spinal epidural abscess diagnosis, including a post-void bladder residual volume.
4. Select appropriate antibiotics for empiric treatment of spinal epidural abscess depending on patient presentation.
5. Disposition the patient to appropriate inpatient care.

**Educational Methods:**

The authors conducted this simulation case with a standardized patient. We encourage inclusion of a standardized patient versus a mannequin to provide appropriate motor and sensory exams. For those without a standardized patient program, the authors suggest utilizing a faculty member as the patient. Regardless of individual used, it is strongly recommended that facilitators rehearse the case with the individual in the patient role ahead of time in order to ensure that their performance reflects an accurate neurologic exam. A debriefing session and small-group discussion followed the simulation to review the clinical presentation, diagnosis, management, and treatment of spinal epidural abscesses. This case can also be adapted as an oral boards case.

**Research Methods:**

Residents were provided a survey at the completion of the debriefing session to rate different aspects of the simulation, as well as to provide qualitative feedback on the scenario. This survey is specific to our institution’s simulation center.

**Results:**

While qualitative feedback from the residents was positive, it was viewed as a straightforward case. Our initial presenting symptom was difficulty ambulating with a fever at home, if asked. The residents appreciated performing a neurologic exam on a standardized patient versus attempting this on a mannequin.

Our simulation center’s feedback form is based on the Center of Medical Simulation’s Debriefing Assessment for Simulation in Healthcare (DASH) Student Version Short Form with the inclusion of required qualitative feedback if an element was scored less than a 6 or 7. This session received all 7 scores (extremely effective/outstanding) other than one 5 score for the element assessing if the instructor set the stage for an engaging learning experience. The learner’s feedback for this 5 score was “kinda went right into the case which was ok.” Our form also includes an area for general feedback about the case at the end. Comments included “Great sim. Expert case writing,” “Fun case and learned a lot,” and “Great case! Appreciated feedback on consulting and the difficult consultant situation.”

**Discussion:**

This is a cost-effective method for reviewing epidural abscess. We chose a chief complaint and history that was slightly atypical from “classic” presentations, but not so esoteric that the residents felt cheated at the end of the scenario. When using a standardized patient in a scenario that may involve a sensitive physical exam, we review with learners and the standardized patient what expectations are during the pre-brief session. For example, residents may say, “we would like to check to see if rectal tone is intact,” and then the standardized patient would verbalize back the expected physical exam findings.

**Topics:**

Medical simulation, spinal epidural abscess, spinal cord compression, infectious disease.

## USER GUIDE

[Table t1-jetem-5-1-s26]

**Table t1-jetem-5-1-s26:** 

List of Resources:	
Abstract	26
User Guide	29
Instructor Materials	31
Operator Materials	41
Standardized Patient Script	44
Debriefing and Evaluation Pearls	46
Simulation Assessment	48

**Learner Audience:**

Medical students, interns, junior residents, senior residents

**Time Required for Implementation:**

Instructor Preparation: 30 minutes

Time for case: 20 minutes

Time for debriefing: 30 minutes

**Recommended Number of Learners per Instructor:**

4

**Topics:**

Medical simulation, spinal epidural abscess, spinal cord compression, infectious disease.

**Objectives:**

After this simulation case, learners will be able to diagnose and manage patients with spinal epidural abscesses. Specifically, learners will be able to:

1. Obtain a detailed history, including past infectious, surgical, procedural and social history to evaluate for epidural abscess risk factors.
2. Describe clinical signs and symptoms of spinal epidural abscesses and understand that initial clinical presentations can be variable.
3. Perform a focused neurological exam including evaluation of motor, sensory, reflexes, and rectal tone.
4. Order appropriate laboratory testing and imaging modalities for spinal epidural abscess diagnosis, including a post-void bladder residual volume.
5. Select appropriate antibiotics for empiric treatment of spinal epidural abscess depending on patient presentation.
6. Disposition the patient to appropriate inpatient care.

### Linked objectives and methods

Spinal epidural abscess is a rare diagnosis with potential for severe neurological sequelae. This is a difficult diagnosis to make due to the frequent lack of classic signs and symptoms. Thus, learners should obtain a detailed history and physical to identify risk factors (objective 1). By understanding the potential signs and symptoms that can present with spinal epidural abscess, the diagnostician can determine from the initial evaluation if spinal epidural abscess should be included in the working differential diagnosis (objective 2). During the patient’s evaluation, learners should perform a focused, yet thorough, neurological exam (objective 3). If during this evaluation the patient is identified to have bladder dysfunction, the learner should measure bladder volume (objective 4). Once suspicion for spinal epidural abscess has been identified, learners should order appropriate testing looking for leukocytosis and elevated inflammatory markers, and emergent MRI imaging (objective 4). More importantly, no delay should be made in obtaining blood cultures and administering empiric antibiotics (objective 5). Patients should be admitted to inpatient care (objective 6) and a spinal surgical service should be consulted for further treatment.

### Recommended pre-reading for instructor

Instructors should review literature covering spinal epidural abscess pathogenesis, epidemiology, risk factors, clinical signs and symptoms, management, and treatment. We recommend that instructors review Darouiche’s 2006 article “Spinal Epidural Abscess” in the *New England Journal of Medicine*.

Other suggested reading includes the materials listed below under “References/suggestions for further reading.”

### Results and tips for successful implementation

This simulation case was written for the emergency medicine setting but can be applied in the urgent care and outpatient office settings. We recommend that a standardized patient be utilized as the patient in order to provide a neurological exam for learners. We conducted a pilot session of this simulation case in January 2019 with approximately 10 emergency medicine residents who ranged from training levels of post-graduate year (PGY)1-3. After completion of the pilot session, we recommend providing limited initial presenting symptoms to better reflect the variable presentation and diagnostic difficulty of this diagnosis. Participant feedback was generally positive during post-session feedback.

## Supplementary Information





























## Figures and Tables

**Table t2-jetem-5-1-s26:** 

#1	Magnetic resonance imaging (MRI) lumbar spine
#2	Chest radiograph (CXR)
#3	Post-void bladder scan
#4	Electrocardiogram (EKG)
#5	Complete blood count (CBC)
#6	Basic metabolic panel (BMP)
#7	Erythrocyte sedimentation rate (ESR)
#8	C-reactive protein (CRP)
#9	Coagulation panel
#10	Beta-human chorionic gonadotropin (β-hCG)
#11	Point-of-care glucose
#12	Lactic acid
#13	Urinalysis
#14	Urine toxicology screen

**Table t3-jetem-5-1-s26:** 

**Vitals:**	Heart Rate (HR) 95	Blood Pressure (BP) 116/89	Respiratory Rate (RR) 12
	Temperature (T) 99.5°F	Oxygen Saturation (O_2_Sat) 98% on room air	
	Weight (Wt) 50 kg		

**Figure f1-jetem-5-1-s26:**
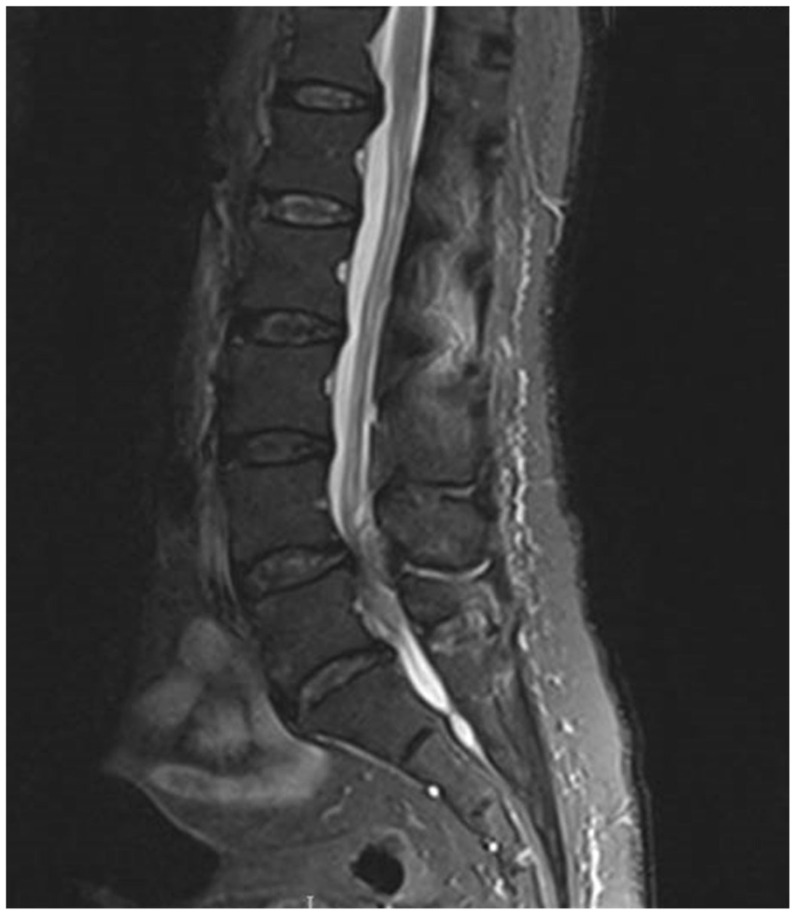


**Figure f2-jetem-5-1-s26:**
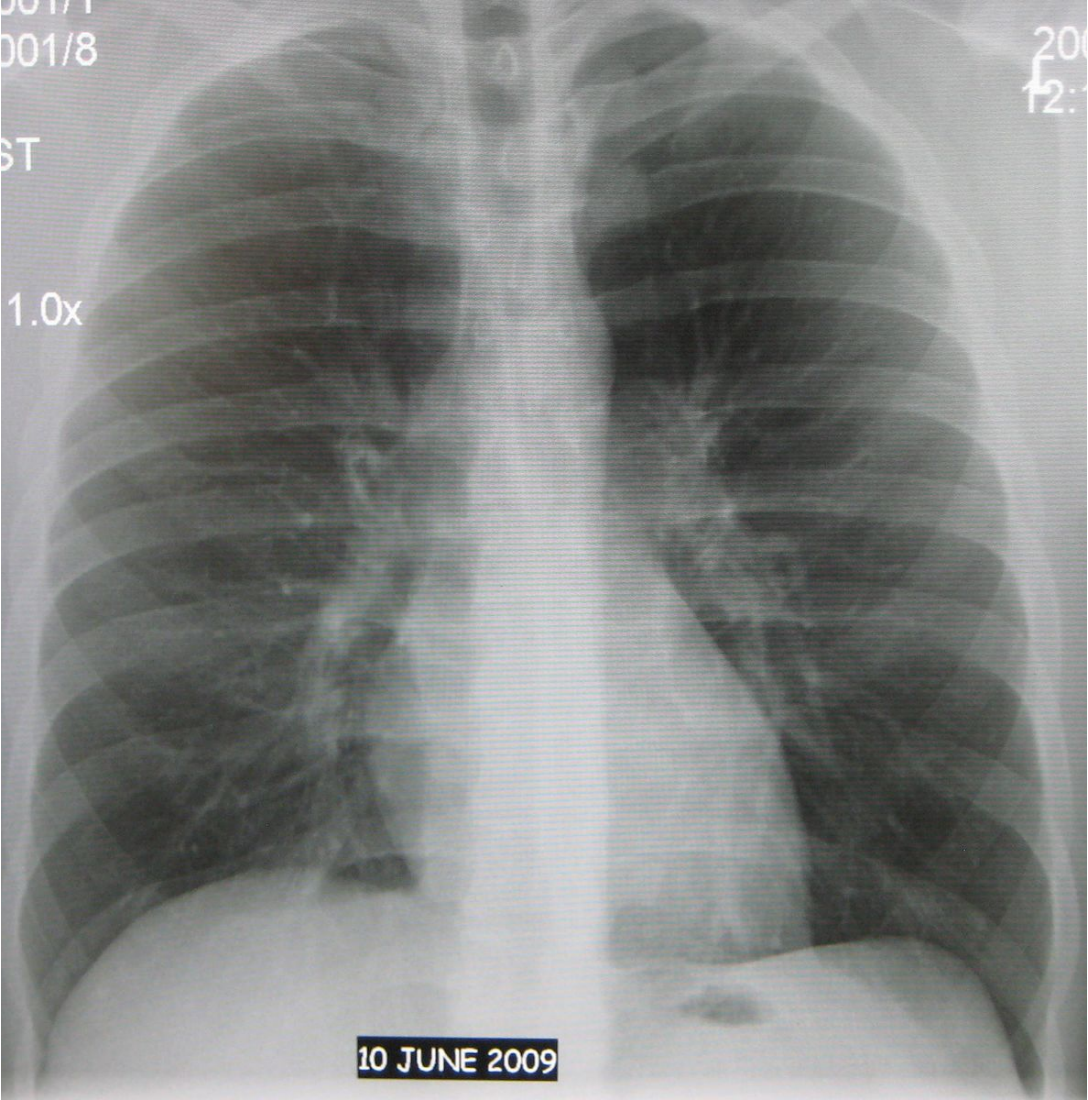


**Table t4-jetem-5-1-s26:** 

*Post-void residual bladder volume*	700 mL

**Figure f3-jetem-5-1-s26:**
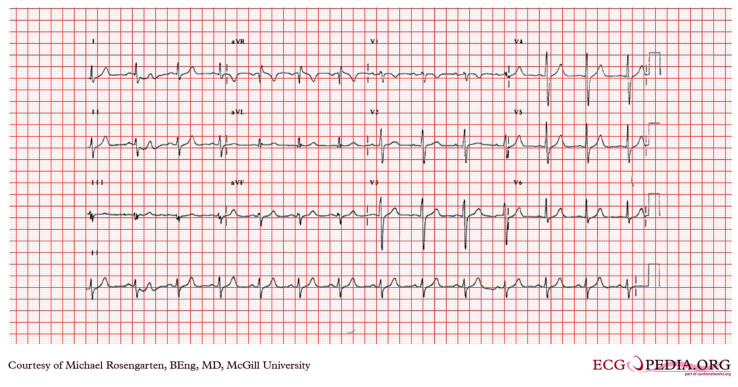


**Table t5-jetem-5-1-s26:** 

*Complete blood count (CBC)*	
White blood count (WBC)	13.8 x1000/mm^3^
Hemoglobin (Hgb)	11.8 g/dL
Hematocrit (HCT)	31.0%
Platelet (Plt)	268 x1000/mm^3^
Segs:	72%
Bands:	8%

**Table t6-jetem-5-1-s26:** 

*Basic metabolic panel (BMP)*	
Sodium	134 mEq/L
Chloride	101 mEq/L
Potassium	4.6 mEq/L
Bicarbonate (HCO_3_)	18 mEq/L
Blood Urea Nitrogen (BUN)	16 mg/dL
Creatine (Cr)	0.98 mg/dL
Glucose	118 mg/dL

**Table t7-jetem-5-1-s26:** 

*Erythrocyte sedimentation rate (ESR)*	74 mm/hr

**Table t8-jetem-5-1-s26:** 

*C reactive protein (CRP)*	28 mm/dL

**Table t9-jetem-5-1-s26:** 

*Coagulation panelz*	
Prothrombin Time (PT)	12 seconds
International Normalized Ratio (INR)	1.0
Partial Thromboplastin Time (PTT)	33 seconds

**Table t10-jetem-5-1-s26:** 

*Qualitative Beta-HCG*	Negative

**Table t11-jetem-5-1-s26:** 

*Point-of-care glucose*	108 mg/dL

**Table t12-jetem-5-1-s26:** 

*Lactic Acid*	2.5 mmol/L

**Table t13-jetem-5-1-s26:** 

*Urinalysis (UA)*	
Leukocyte esterase	negative
Nitrites	negative
Blood	none
Protein	2+
Ketones	none
Glucose	none
Color	dark yellow
White blood cells (WBC)	0–5 WBCs/high powered field (HPF)
Red blood cells (RBC)	0–5 RBCs/HPF
Squamous epithelial cells	0–5 cells/HPF
Specific gravity	1.020

**Table t14-jetem-5-1-s26:** 

*Urine Toxicology Screen*	
Amphetamines	Negative
Barbiturates	Negative
Benzodiazepines	Negative
Cocaine	Negative
Methadone	Negative
Opiates	Negative
Oxycodone	Negative
PCP	Negative
THC	Negative

**Table t15-jetem-5-1-s26:** SIMULATION EVENTS TABLE

Minute (state)	Participant action/trigger	Patient status (simulator response) & operator prompts	Monitor display(vital signs)
0:00 (Baseline)	Participants should place the patient on the monitor and obtain vital signs.	Patient is lying left lateral recumbent on emergency department bed. Nursing states patient arrives from home by EMS.	T 99.5°F HR 95 BP 116/89 RR 12 O_2_ sat 98% RA
02:00	Participants should obtain history, order point-of-care glucose, and perform physical exam. The team should order IV access and labs.	Patient repeatedly asks for pain medications, including stating “Can I have something for pain? hydromorphone has helped before.” If participants express intent to perform a genitourinary exam, patient will verbalize findings, including confirming perineal numbness and decreased rectal tone. If participants plan to discharge patient, patient will be unsteady upon trying to get out of bed and have to be steadied by the nurse.	T 99.5°F HR 95 BP 116/89 RR 12 O_2_ sat 98% RA
06:00	MRI should be ordered. Blood work, urine studies, post-void bladder residual volume are available. Participants should conclude that patient likely has a spinal epidural abscess leading to spinal compression. Antibiotics should be ordered.	If participants do not order a post-void bladder residual volume, patient may say, “I can’t make myself pee.” If participants do not recognize epidural spinal abscess leading to spinal compression and have not ordered an MRI, then patient may state that she has had weakness and can’t walk. If participants decide to order a urinary catheter and then discharge the patient, patient will be unsteady upon trying to get out of bed and have to be steadied by the nurse. Patient may ask, “How can I go home when I can’t walk?!”	T 99.5°F HR 95 BP 116/89 RR 12 O_2_ sat 98% RA
09:00	If antibiotics have not been given, participants should respond to change in vital signs as follows: order IV fluid resuscitation, administer antipyretic, and start IV antibiotics.	If antibiotics are already given, proceed to 12:00 (A). If antibiotics are not given, then vital signs will worsen. If the team does not ask for a repeat temperature, the nurse will put their hand on the patient and say “You feel warmer than you did before.”	With IV antibiotics: T 100°F HR 95 BP 116/89 RR 12 O_2_ sat 98% RA Without IV antibiotics: T 102°F HR 120 BP 100/75 RR 20 O_2_ sat 98% RA
12:00	MRI spine study results available, if ordered. Participants call surgical consultant for potential operative intervention of spinal epidural abscess.	When the orthopaedic or neurosurgery consultant is called, they will state, “I am in the middle of a surgical case, but will be down shortly to evaluate the patient.” They will request imaging (if not yet performed) and supportive care. If the inpatient team is called without consulting neurosurgery or orthopaedic surgery, they will ask for etiology of patient’s urinary retention. Inpatient team will ask for patient’s physical exam, including neurological exam. If participants have not ordered an MRI of the spine by this time, inpatient team will ask for further workup of a possible neurological etiology prior to admission. If participants attempt to admit the patient at this time without having given antibiotics, the inpatient/surgical team will refuse admission, citing multiple vital sign abnormalities and need for further resuscitation	With IV antibiotics: T 100°F HR 95 BP 116/89 RR 12 O_2_ sat 98% RA Without IV antibiotics by minute 12: T 102°F HR 136 BP 94/70 RR 24 O_2_ sat 98% RA

**Table t16-jetem-5-1-s26:** 

**Critical Actions:** Perform back exam, including palpation of the entire spine and direct visual inspection. Perform neurological exam assessing for motor function, sensation, and reflexes. Obtain post-void residual bladder volume/bladder scan. Order emergent spinal MRI. Administer broad-spectrum antibiotics. Discuss the case with neurosurgery or orthopedic surgery. Admit to inpatient unit.	0:00

**Table t17-jetem-5-1-s26:** 

	Milestone	Did not achieve level 1	Level 1	Level 2	Level 3
1	**Emergency Stabilization (PC1)**	□ Did not achieve Level 1	□ Recognizes abnormal vital signs	□ Recognizes an unstable patient, requiring intervention Performs primary assessment Discerns data to formulate a diagnostic impression/plan	□ Manages and prioritizes critical actions in a critically ill patient Reassesses after implementing a stabilizing intervention
2	**Performance of focused history and physical (PC2)**	□ Did not achieve Level 1	□ Performs a reliable, comprehensive history and physical exam	□ Performs and communicates a focused history and physical exam based on chief complaint and urgent issues	□ Prioritizes essential components of history and physical exam given dynamic circumstances
3	**Diagnostic studies (PC3)**	□ Did not achieve Level 1	□ Determines the necessity of diagnostic studies	□ Orders appropriate diagnostic studies. Performs appropriate bedside diagnostic studies/procedures	□ Prioritizes essential testing Interprets results of diagnostic studies Reviews risks, benefits, contraindications, and alternatives to a diagnostic study or procedure
4	**Diagnosis (PC4)**	□ Did not achieve Level 1	□ Considers a list of potential diagnoses	□ Considers an appropriate list of potential diagnosis May or may not make correct diagnosis	□ Makes the appropriate diagnosis Considers other potential diagnoses, avoiding premature closure
5	**Pharmacotherapy (PC5)**	□ Did not achieve Level 1	□ Asks patient for drug allergies	□ Selects an medication for therapeutic intervention, consider potential adverse effects	□ Selects the most appropriate medication and understands mechanism of action, effect, and potential side effects Considers and recognizes drug-drug interactions
6	**Observation and reassessment (PC6)**	□ Did not achieve Level 1	□ Reevaluates patient at least one time during case	□ Reevaluates patient after most therapeutic interventions	□ Consistently evaluates the effectiveness of therapies at appropriate intervals
7	**Disposition (PC7)**	□ Did not achieve Level 1	□ Appropriately selects whether to admit or discharge the patient	□ Appropriately selects whether to admit or discharge Involves the expertise of some of the appropriate specialists	□ Educates the patient appropriately about their disposition Assigns patient to an appropriate level of care (ICU/Tele/Floor) Involves expertise of all appropriate specialists
9	**General Approach to Procedures (PC9)**	□ Did not achieve Level 1	□ Identifies pertinent anatomy and physiology for a procedure Uses appropriate Universal Precautions	□ Obtains informed consent Knows indications, contraindications, anatomic landmarks, equipment, anesthetic and procedural technique, and potential complications for common ED procedures	□ Determines a back-up strategy if initial attempts are unsuccessful Correctly interprets results of diagnostic procedure
20	**Professional Values (PROF1)**	□ Did not achieve Level 1	□ Demonstrates caring, honest behavior	□ Exhibits compassion, respect, sensitivity and responsiveness	□ Develops alternative care plans when patients’ personal beliefs and decisions preclude standard care
22	**Patient centered communication (ICS1)**	□ Did not achieve level 1	□ Establishes rapport and demonstrates empathy to patient (and family) Listens effectively	□ Elicits patient’s reason for seeking health care	□ Manages patient expectations in a manner that minimizes potential for stress, conflict, and misunderstanding. Effectively communicates with vulnerable populations, (at risk patients and families)
23	**Team management (ICS2)**	□ Did not achieve level 1	□ Recognizes other members of the patient care team during case (nurse, techs)	□ Communicates pertinent information to other healthcare colleagues	□ Communicates a clear, succinct, and appropriate handoff with specialists and other colleagues Communicates effectively with ancillary staff 1

## INSTRUCTOR MATERIALS

**Case Title:** Spinal Epidural Abscess

**Case Description & Diagnosis (short synopsis):** Patient is a 45-year-old female with history of type 1 diabetes mellitus presents with urinary retention and fatigue for two days. Upon presentation, patient is tearful, distressed and reporting generalized pain. Participants should clarify that patient’s complaint of fatigue is due to feeling weak. A detailed physical exam will reveal upper extremity track marks consistent with IV drug use and lower extremity hyporeflexia, paresthesias, and weakness. For diagnostic workup, blood work, a post-void residual volume, and an emergent lumbar MRI with contrast should be obtained. Results will demonstrate leukocytosis, increased post-void bladder volume and a spinal MRI consistent with a spinal epidural abscess. Appropriate antibiotics and pain medication should be ordered and patient case discussed with spinal surgery consultant. Patient should ultimately be admitted to inpatient care.

**Equipment or Props Needed:**

Upper extremity moulage consistent with track marks

For lower-cost utilization, can use makeup and a small damp makeup brush to apply faint dark red color in small spots in a linear fashion or in a cluster over the patient’s natural upper extremity vasculature.

Cardiac monitor

Pulse oximetry

Temperature probe

Angiocatheters for peripheral intravenous access (18 to 22 gauge)

Intravenous (IV) pole

Ultrasound for bladder volume assessment

Simulated medications with labeling: acetaminophen, vancomycin, ceftriaxone, hydromorphone, morphine

**Confederates needed:**

Patient and primary nurse. Facilitators running the simulation may call in as orthopaedic or neurosurgical consultants and the hospitalist.

**Stimulus Inventory:** [Table t2-jetem-5-1-s26]

**Background and brief information:** Patient called Emergency Medical Services (EMS) for fatigue and urinary retention. Patient presents to the emergency department with paramedics.

**Initial presentation:** Patient is a 45-year-old female with history of type 1 diabetes mellitus who presents via EMS from home with urinary retention and fatigue. Patient is lying on left side, tearful, and able to converse. Patient reports history of chronic pain and asks repeatedly for pain medication.

**How the scenario unfolds:** Patient is a 45-year-old female who presents via EMS from home with urinary retention, fatigue, and generalized pain. Participants should perform a physical exam to identify signs of IV drug use and neurological deficits. A post-void residual bladder volume should be obtained, showing urinary retention. The participants should order blood work for infectious workup, initiate antibiotics, and obtain an emergent spinal MRI showing spinal epidural abscess. If antibiotics are not initiated by the 9-minute mark, patient will begin to have progressively worsening tachycardia, hypotension, and will develop a fever. If participants attempt to discharge patient during scenario, patient will be unable to safely ambulate.

**Critical actions:**

1. Perform back exam, including palpation of the entire spine and direct visual inspection.
2. Perform neurological exam assessing for motor function, sensation, and reflexes.
3. Obtain post-void residual bladder volume/bladder scan.
4. Order emergent spinal MRI.
5. Administer broad spectrum antibiotics.
6. Discuss the case with neurosurgery or orthopaedic surgery.
7. Admit to inpatient unit.

**Case Title:** Spinal Epidural Abscess

**Chief Complaint:** Urinary retention and fatigue[Table t3-jetem-5-1-s26]

**General Appearance:** Lying on left side in bed, tearful

**Primary Survey:**

- **Airway:** Intact
- **Breathing:** Clear to auscultation
- **Circulation:** Regular rate and rhythm, no murmurs, 2+ distal pulses

**History:**

- **History of present illness:** Patient is a 45-year-old female who presents via EMS from home. She has had fatigue for two days. If asked specifically, she feels like she can’t fully empty her bladder despite the need to do so and she often urinates small amounts on herself whenever she coughs or sneezes. Patient appears distressed, tearful, and repeatedly reports chronic 10/10 generalized pain. Patient called EMS today because she could not get out of bed. Denies falls, trauma, or recent diarrhea.
- **Past medical history**: Type 1 diabetes mellitus
- **Past surgical history:** No prior surgeries
- **Medications:** Insulin
- **Allergies:** None
- **Social history:** When initially asked about alcohol, smoking, or drugs, patient refuses to answer, saying “I don’t see how that is important right now. Can’t you see I’m in pain?” If learners then explain why they are asking social history questions, the patient will admit to 2–3 alcoholic drinks a week, smoking tobacco cigarettes, and will shrug and nod affirmatively if asked about intravenous drug use (but still will not divulge further details regarding this).
- **Family history:** Type 2 diabetes (maternal grandparents)

**Secondary Survey/Physical Examination:**

- **General appearance:** Patient is lying on her left side. She appears distressed and tearful. She repeatedly asks for pain medication. Patient is otherwise able to converse appropriately.
- **HEENT:**
  ○ **Head:** within normal limits
  ○ **Eyes:** within normal limits
  ○ **Ears:** within normal limits
  ○ **Nose:** within normal limits
  ○ **Throat:** within normal limits
- **Neck:** within normal limits
- **Heart:** Normal rate and rhythm, no murmurs
- **Lungs:** Clear lung sounds
- **Abdominal/GI:** Suprapubic discomfort upon palpation without guarding, rebound, or rigidity
- **Genitourinary:** Palpable bladder appreciated extending caudally 2/3 up to the umbilicus
- **Rectal:** (verbalized by the standardized patient): Decreased rectal tone and decreased perianal sensation
- **Extremities:** Track marks noted diffusely over the bilateral upper extremities without superimposed cellulitis
- **Back:** No skin changes or deformities noted other than upper extremity track marks as above. Diffuse tenderness over the lower lumbar spine. No costovertebral angle tenderness.
- **Neuro:** Glasgow coma scale (GCS) 15 (eyes 4, verbal 5, motor 6). Cranial nerves intact. 5/5 symmetric upper extremity motor strength with shoulder shrug, elbow flexion and extension, wrist flexion, wrist extension, anterior interosseus muscles (AIN), posterior interosseous muscles (PIN), and intrinsics. 5/5 hip flexion bilaterally. 3/5 motor strength with knee flexion, knee extension, plantar flexion, dorsiflexion, and extensor hallucis longus bilaterally. Sensation is intact over upper extremity dermatomes bilaterally. Decreased sensation diffusely over bilateral lower extremities, but particularly decreased in the L4 distribution from the anterior knee and extending medially and distally to the medial malleolus. Symmetric 1+ patellar and Achilles reflexes. Intact finger-to-nose testing bilaterally. Heel-to-shin maneuvers unable to be completed due to lower extremity weakness.
- **Skin:** Track marks over bilateral upper extremities, as above
- **Lymph:** within normal limits
- **Psych:** Anxious, denies suicidal ideation

**Results:**

*MRI lumbar spine*

Chan JJ, Oh JJ. MRI of the lumbar spine of a 57-year-old woman: The collection in the posterior epidural space and the posterior disc bulge is seen compressing the thecal sac and the cauda equina. In: Wikimedia Commons.

https://commons.wikimedia.org/wiki/File:MRI_of_the_lumbar_spine_with_abscess_in_the_posterior_epidural_space,_causing_cauda_equina_syndrome.jpg. CC-BY 4.0.[Fig f1-jetem-5-1-s26]

*Chest Radiograph (CXR)*

Heilman, J. Normal AP chest xray. In: Wikimedia Commons.

https://commons.wikimedia.org/wiki/File:Normal_AP.JPG. CC BY 3.0.[Fig f2-jetem-5-1-s26][Table t4-jetem-5-1-s26]

*Electrocardiogram (EKG)*

Rosengarten, M. Below is the final EKG after the patient reverted to sinus rhythm. In: Wikimedia Commons.

https://commons.wikimedia.org/wiki/File:E3594_(CardioNetworks_ECGpedia).jpg. CC BY-SA 3.0.[Fig f3-jetem-5-1-s26][Table t5-jetem-5-1-s26][Table t6-jetem-5-1-s26][Table t7-jetem-5-1-s26][Table t8-jetem-5-1-s26][Table t9-jetem-5-1-s26][Table t10-jetem-5-1-s26][Table t11-jetem-5-1-s26][Table t12-jetem-5-1-s26][Table t13-jetem-5-1-s26][Table t14-jetem-5-1-s26]

## OPERATOR MATERIALS

[Table t15-jetem-5-1-s26]

### Diagnosis

Spinal Epidural Abscess

### Disposition

Admit to inpatient unit

## STANDARDIZED PATIENT MATERIALS

### Epidural Abscess Standardized Patient Script

Positioning- lying curled up on your left side, intermittently tearful

Patient – intermittent statements

- “My back hurts so bad.”
- “Can I have something for pain?”
- “Hydromorphone has helped me before.”

History of present illness

- Felt “really run down” for the past two days.
- You have had trouble standing and walking for the past two days – “My legs just won’t work.”
  ○ Now you have to hold on to things to get around or hold on to someone for support.
  ○ Normally ambulates without any problems.
  ○ If asked, you have not had diarrhea recently.
- Normally has chronic back pain “all over” and you’re not sure if it is worse today. “It’s always a 10/10.”
  ○ Takes tramadol, ibuprofen, and acetaminophen as needed for pain.
  ○ No recent falls, injuries, or trauma.
- If asked, there has been a fever up to 100.8°F intermittently for the past three days.
- If asked, you cannot fully empty your bladder despite the need to do so and you are now urinating small amounts on yourself when you cough or sneeze.

Past medical history

- Type 1 diabetes, for which you take sliding scale insulin.
- No previous surgeries.
- You are not allergic to anything.
- If they initially ask about alcohol, smoking, or drugs, you will initially refuse to answer, saying “I don’t see how that is important right now. Can’t you see if I’m in pain?”
  ○ If learners then explain why they are asking social history questions, you will confirm that you drink 2–3 alcoholic drinks a week and smoke tobacco cigarettes.
  ○ If they ask about intravenous drug use, shrug and nod affirmatively, but when asked details, mention again that your back pain really hurts right now.
- Various family members (if asked, your maternal grandparents) have had type 2 diabetes.

Physical exam

- Your midback and low back will significantly hurt, even if they press lightly. Any movement will make you grimace in pain.
- When they press over your lower central abdomen (2/3 up to your umbilicus/belly button from your pubic bone in the front), you’ll say “Careful, that makes me feel like I have to pee.”
- Your strength is normal in your arms.
- If they ask about your track marks, say “oh, that’s from my insulin.”
- In your legs, you can weakly do what they ask (lift your leg, bend your knee, straighten your knee, toes up, toes down, big toe up) if it is against gravity, or when you’re lying on your side without them putting any pressure on your legs for resistance. Start crying and ask “what’s wrong with my legs?”
- If they ask you to stand up or walk, you will almost fall while getting out of bed to stand and have to be steadied by the nurse.
  ○ If learners ask if you have trouble walking/moving your legs due to weakness or pain, become tearful and say “Both! My back always hurts, but my legs just aren’t working right.”
  ○ If they ask about sensation in your legs, it’ll be less than normal everywhere, but almost completely numb over the middle of your kneecaps, the middle lower legs, and your inner ankles bilaterally.
- If the learners ask about rectal tone, you can verbalize “Rectal tone is intact.”

Workup

- If the learners order a foley catheter, say “Whoa, why are you ordering that?” Then agree to have it placed once learners explain why it is needed.
- If the learners discharge you, you will almost fall while getting out of bed to stand and have to be steadied by the nurse. You will then say “How can I go home when I can’t walk?!”
- If the learners do not explain what is going on after the MRI test, ask them “what did the tests show?”

## DEBRIEFING AND EVALUATION PEARLS

### Spinal Epidural Abscess

#### Risk Factors

Various risk factors have been associated with development of epidural abscesses, as follows 2,5:

1. Preexisting conditions including immunocompromising conditions, diabetes mellitus, human immunodeficiency virus (HIV), alcoholism, chronic liver disease, chronic kidney disease
2. Procedures including epidural anesthesia, spinal surgery, spinal procedures, acupuncture, tattooing
3. Implantations including spinal hardware and indwelling vascular catheters
4. Intravenous drug use
5. Infection including osteomyelitis, sepsis, bacteremia, soft tissue infection
6. Spinal fracture or deformities

#### Symptoms/Findings

The diagnosis of epidural abscess is difficult due to lack of disease-defining symptoms or clinical findings. Epidural abscesses are classically associated with fever, back pain, and neurological deficits; however, patients seldomly present with all these symptoms.^2^,^5^ Some patients may not initially present with fever.^4^ Thus, awareness and suspicion for this diagnosis is necessary. Practitioners should consider obtaining complete blood count to evaluate for leukocytosis and ordering inflammatory markers. Unfortunately, leukocytosis is only found in about two-thirds of spinal epidural abscess cases and elevations of ESR or CRP are not specific for this diagnosis.^2^

#### Diagnosis

The imaging modality of choice for diagnosis is MRI with contrast.^2^ Consider imaging multiple areas of the spinal cord given the epidural abscess may extend further than one spinal segment or may involve multiple spinal segments.^4^ Lumbar puncture is not recommended due to risk of infectious spread if the abscess is accessed during the procedure.^4^

#### Other debriefing points

- What imaging modalities would learners pursue if the patient was unable to obtain an MRI due to an incompatible pacemaker or retained metal?
- How did you address analgesia in this patient?
- Tell me about your neurologic exam. Now knowing the diagnosis, would you have performed this neurologic exam differently?
- What would you do if the patient became tearful and asked if they did this to themselves due to the intravenous drug use? What about if the patient wishes to leave against medical advice?
- Ensure that closed-loop communication was used between team members. Reflect on whether closed-loop communication occurred. Discuss why it was or was not used. Discuss how use or lack of closed-loop communication affected outcomes during the case.

## SIMULATION ASSESSMENT

*Spinal Epidural Abscess*

Learner: _________________________________________

***Assessment Timeline***

This timeline is to help observers assess their learners. It allows observer to make notes on when learners performed various tasks, which can help guide debriefing discussion.[Table t16-jetem-5-1-s26]

**Critical Actions:**

□ Perform back exam, including palpation of the entire spine and direct visual inspection.
□ Perform neurological exam assessing for motor function, sensation, and reflexes.
□ Obtain post-void residual bladder volume/bladder scan.
□ Order emergent spinal MRI.
□ Administer broad-spectrum antibiotics.
□ Discuss the case with neurosurgery or orthopaedic surgery.
□ Admit to inpatient unit.

**Summative and formative comments:**

**Milestones assessment:** [Table t17-jetem-5-1-s26]

## References

[1] Tanski ME Ma OJ Central nervous system and spinal infections Tintinalli JE Stapczynski JS Ma OJ Yealy DM Meckler GD Cline DM Tintinalli’s Emergency Medicine: A Comprehensive Study Guide 8th ed New York, NY McGraw-Hill Education 2016 accessmedicine.mhmedical.com/content.aspx?aid=1121512340 Accessed April 15, 2019

[2] Darouiche RO Spinal epidural abscess N Engl J Med 2006 355 19 2012 2020 10.1056/NEJMra055111 17093252

[3] Ropper AE Ropper AH Acute spinal cord compression N Engl J Med 2017 376 14 1358 1369 10.1056/NEJMra1516539 28379788

[4] Sexton DJ Sampson JH Spinal epidural abscess Calderwood SB UpToDate Waltham, Mass UpToDate https://www.uptodate.com/contents/spinal-epidura-labscess. Updated Jun 25, 2019 Accessed Dec 27, 2019

[5] Davis DP Salazar A Chan TC Vilke GM Prospective evaluation of a clinical decision guideline to diagnose spinal epidural abscess in patients who present to the emergency department with spine pain J Neurosurg Spine 2011 14 6 765 770 10.3171/2011.1.SPINE1091 21417700

[6] Boody BS Jenkins TJ Maslak J Hsu WK Patel AA Vertebral osteomyelitis and spinal epidural abscess: an evidence-based review J Spinal Disord Tech 2015 Jul 28 6 E316 27 10.1097/BSD.0000000000000294 26079841

[7] Babic M Simpfendorfer CS Berbari EF Update on spinal epidural abscess Curr Opin Infect Dis 2019 Jun 32 3 265 271 10.1097/QCO.0000000000000544 31021957

