# Supplementary figures and images for: Spinal Epidural Abscess
Source: J Educ Teach Emerg Med. 2020 Jan 15;5(1):S26–52. doi: 10.21980/J8T938 (PMC10332528; doi:10.21980/J8T938)

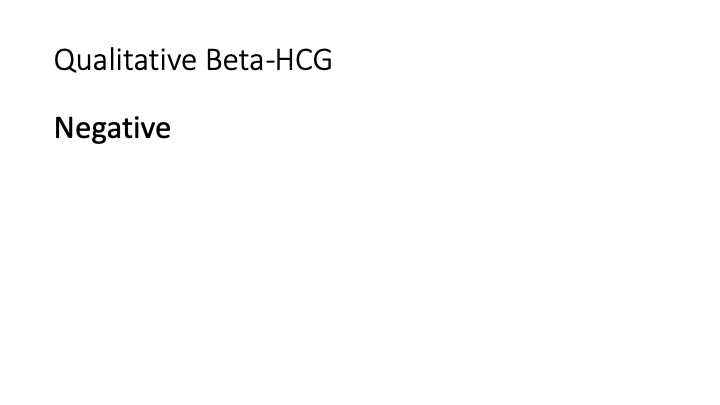

Supplement: Supplementary file 1 [file jetem-5-1-s26-supp1.jpeg]

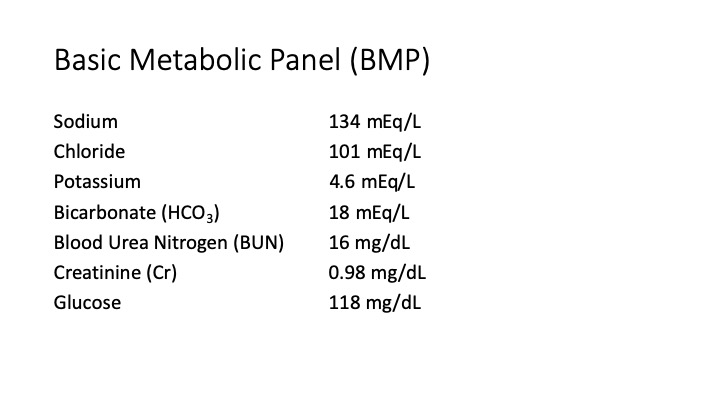

Supplement: Supplementary file 2 [file jetem-5-1-s26-supp2.jpeg]

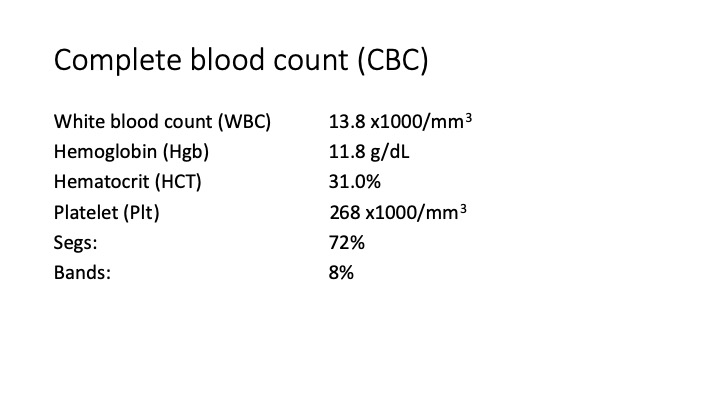

Supplement: Supplementary file 3 [file jetem-5-1-s26-supp3.jpeg]

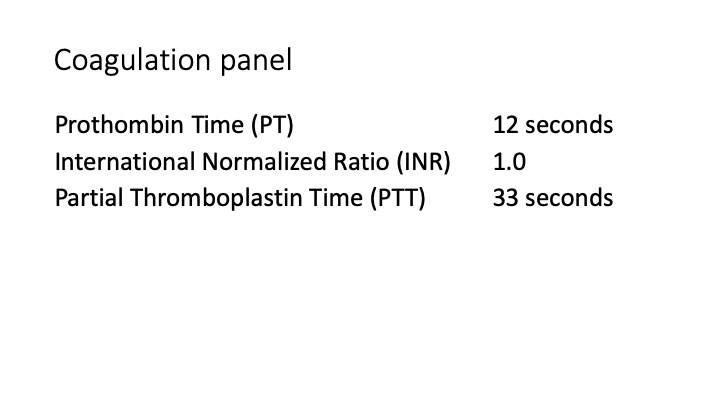

Supplement: Supplementary file 4 [file jetem-5-1-s26-supp4.jpeg]

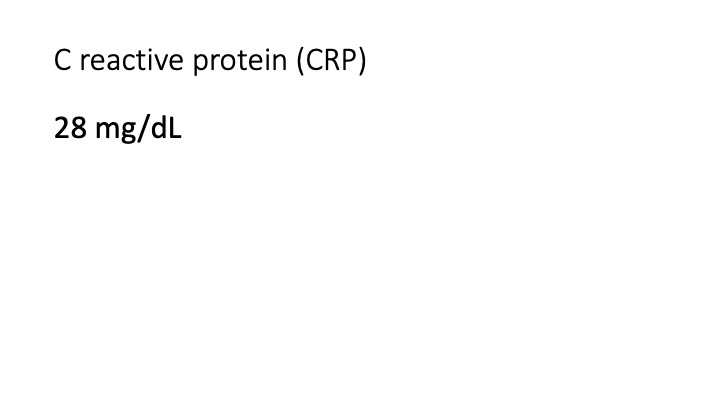

Supplement: Supplementary file 5 [file jetem-5-1-s26-supp5.jpeg]

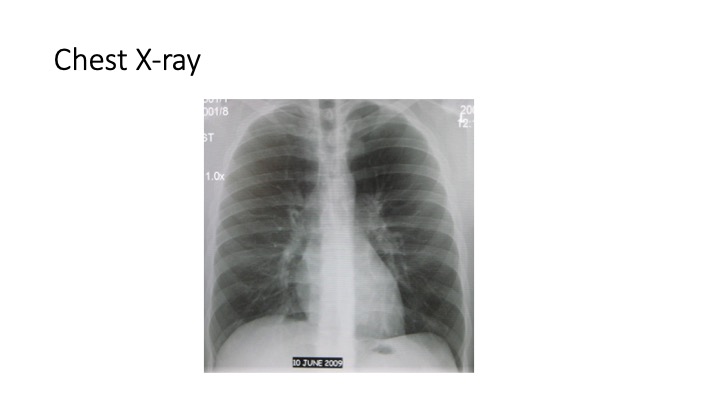

Supplement: Supplementary file 6 [file jetem-5-1-s26-supp6.jpeg]

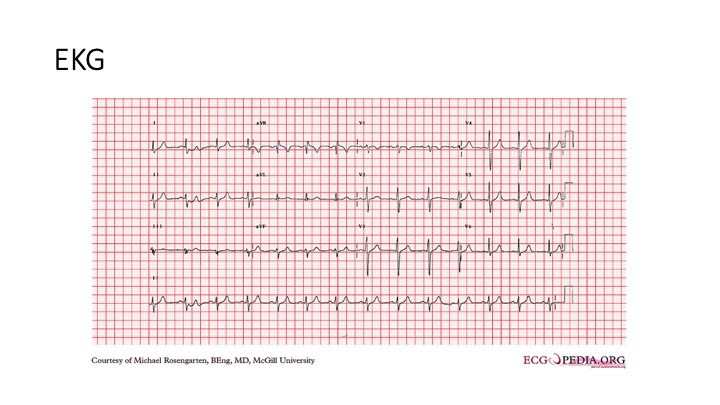

Supplement: Supplementary file 7 [file jetem-5-1-s26-supp7.jpeg]

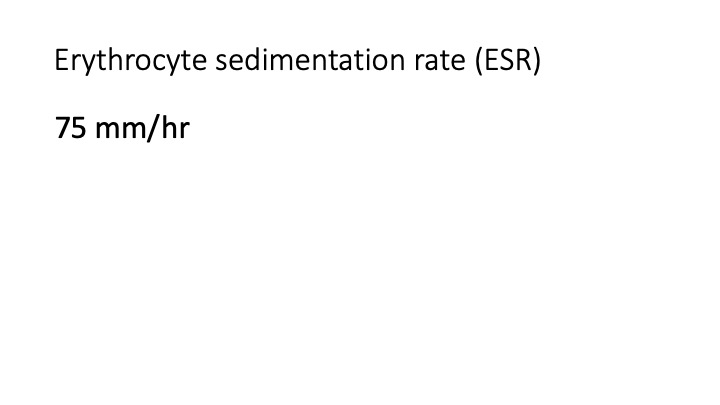

Supplement: Supplementary file 8 [file jetem-5-1-s26-supp8.jpeg]

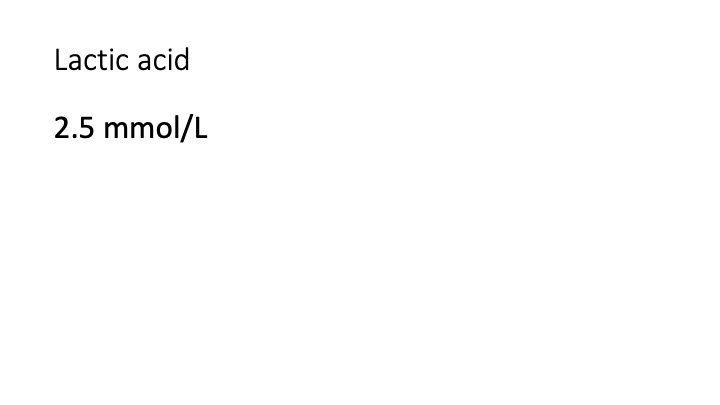

Supplement: Supplementary file 9 [file jetem-5-1-s26-supp9.jpeg]

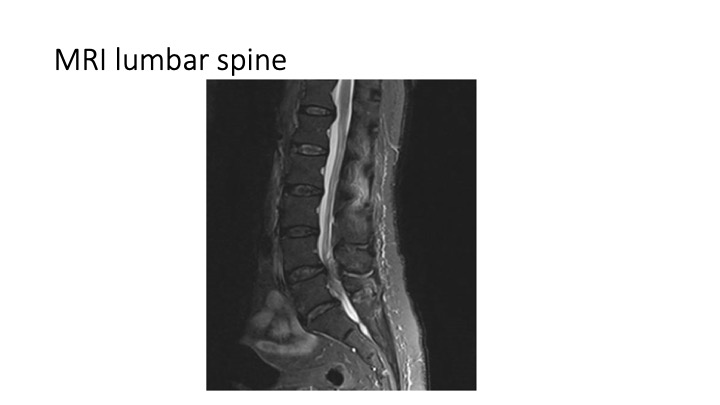

Supplement: Supplementary file 10 [file jetem-5-1-s26-supp10.jpeg]

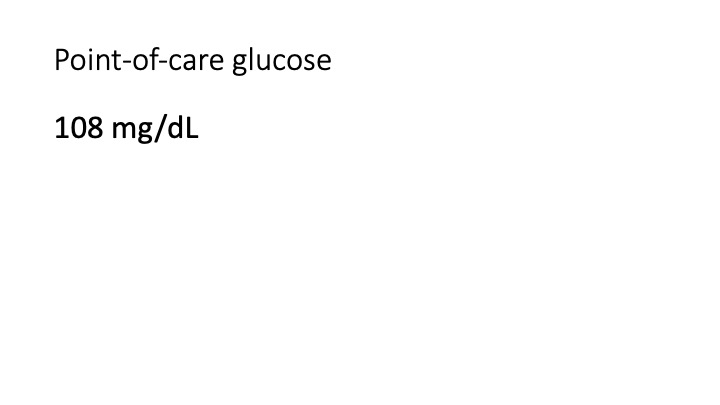

Supplement: Supplementary file 11 [file jetem-5-1-s26-supp11.jpeg]

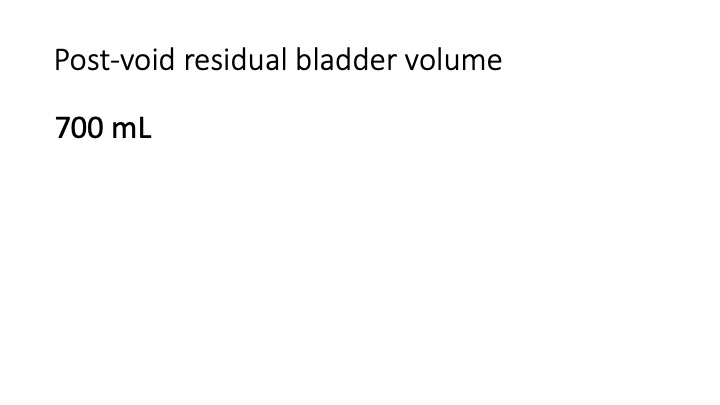

Supplement: Supplementary file 12 [file jetem-5-1-s26-supp12.jpeg]

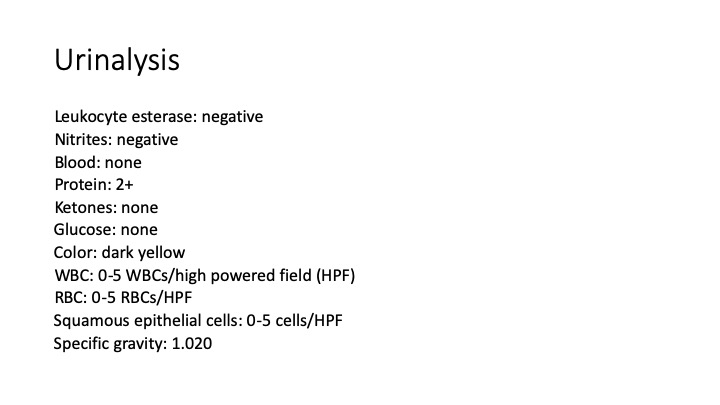

Supplement: Supplementary file 13 [file jetem-5-1-s26-supp13.jpeg]

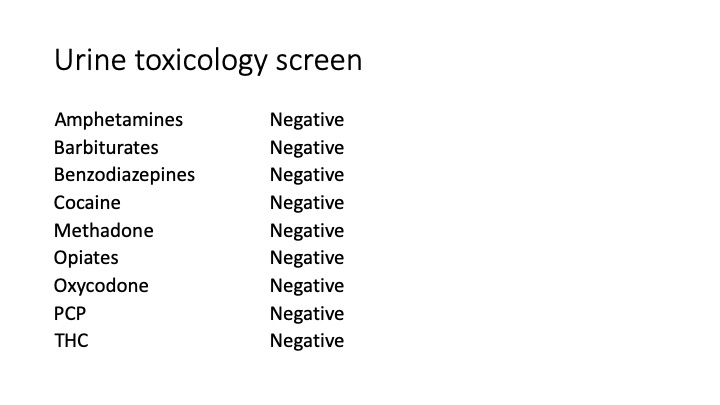

Supplement: Supplementary file 14 [file jetem-5-1-s26-supp14.jpeg]
